# Emerging ensembles of kinetic parameters to characterize observed metabolic phenotypes

**DOI:** 10.1186/s12859-018-2181-7

**Published:** 2018-07-09

**Authors:** Riccardo Colombo, Chiara Damiani, David Gilbert, Monika Heiner, Giancarlo Mauri, Dario Pescini

**Affiliations:** 1 Department of Informatics, Systems and Communication, University of Milan - Bicocca, Viale Sarca, 336, Milan, 20126 Italy; 2 SYSBIO - Centre of Systems Biology, Piazza della Scienza, 2, Milan, 20126 Italy; 3 Department of Statistics and Quantitative Methods, University of Milan - Bicocca, Via Bicocca degli Arcimboldi, 8, Milan, 20126 Italy; 4 College of Engineering, Design and Physical Sciences, Brunel University, Middlesex, London, Uxbridg, UB8 3PH UK; 5 Computer Science Department, Brandenburg University of Technology Cottbus-Senftenberg, Walther-Pauer-Str. 2, Cottbus, D-03046 Germany

**Keywords:** Ensembles, Fluxes, Kinetic parameters, Mechanistic simulations, Metabolism, ODEs, Steady state, Systems biology

## Abstract

**Background:**

Determining the value of kinetic constants for a metabolic system in the exact physiological conditions is an extremely hard task. However, this kind of information is of pivotal relevance to effectively simulate a biological phenomenon as complex as metabolism.

**Results:**

To overcome this issue, we propose to investigate emerging properties of ensembles of sets of kinetic constants leading to the biological readout observed in different experimental conditions. To this aim, we exploit information retrievable from constraint-based analyses (i.e. metabolic flux distributions at steady state) with the goal to generate feasible values for kinetic constants exploiting the mass action law. The sets retrieved from the previous step will be used to parametrize a mechanistic model whose simulation will be performed to reconstruct the dynamics of the system (until reaching the metabolic steady state) for each experimental condition. Every parametrization that is in accordance with the expected metabolic phenotype is collected in an ensemble whose features are analyzed to determine the emergence of properties of a phenotype. In this work we apply the proposed approach to identify ensembles of kinetic parameters for five metabolic phenotypes of *E. Coli*, by analyzing five different experimental conditions associated with the ECC2comp model recently published by Hädicke and collaborators.

**Conclusions:**

Our results suggest that the parameter values of just few reactions are responsible for the emergence of a metabolic phenotype. Notably, in contrast with constraint-based approaches such as Flux Balance Analysis, the methodology used in this paper does not require to assume that metabolism is optimizing towards a specific goal.

**Electronic supplementary material:**

The online version of this article (10.1186/s12859-018-2181-7) contains supplementary material, which is available to authorized users.

## Background

Advances in the understanding of biological processes has revealed that living organisms must be analyzed by taking into account the complex network of interactions among different entities such as genes, transcripts, proteins and metabolites in order to decipher emergent behaviors and regulatory processes. In the context of Systems Biology [1], the study of metabolism has received great interest, especially due to the fruitful applications in metabolic engineering [2]. In these studies, metabolic networks are typically represented as hyper-graphs in which nodes denote metabolites and edges indicate reactions [3].

### Omics data in metabolic modeling

High throughput information allowed the generation of detailed genome-scale metabolic reconstructions, defined ad hoc for different cell types (as e.g. unicellular organisms [4], healthy and diseased tissues in mammalian [5]). Despite this, there are still technological hindrances preventing mechanistic simulation of genome-scale metabolic models: currently, simulated temporal dynamics of metabolic concentrations are practical only for small models due to shortage of retrievable parameter values and high computational costs [6].

### Constraint-based methods

The points raised above determine the current strategy in metabolic modeling, namely the exploitation of the so called constraint-based approaches [7]. This modeling framework uses information about the structure of the metabolic network and assumes that internal metabolites reach a steady-state concentration. Even if these approaches neglect the temporal evolution of the system, they can be considered a valid framework to describe metabolism because of experimental studies pointing out that in vivo metabolism reaches the steady state in few seconds [8]. In the context of constraint-based modeling, a metabolic network is usually described as a set of chemical reactions from which it is possible to retrieve the stoichiometric matrix, i.e. the table illustrating changes in metabolites quantities due to the firing of reactions. Moreover constraint-based approaches define the mathematical space containing flux distributions (i.e. flux values for each reaction in the model) that can be reached by the system under different functional states. This “feasible solution space”, is determined by imposing a mass balance constraint, as well as boundaries on fluxes (e.g. to specify their reversibility). Once the stoichiometric matrix and the boundaries are defined, it is possible to assume that the system is optimal toward a given Objective Function (OF) – such as the production of biomass or a given metabolite – to be maximized or minimized. Following this an optimal flux distributions can be calculated by means of optimization techniques such as Flux Balance Analysis (FBA) [9].

Choosing an appropriate formulation of the OF is of paramount importance when conducting FBA, however often its exact formulation is not definable. Moreover, questioning the concept of optimal behavior, recent studies [10] speculate that it is reasonable to assume that the system is found in a sub-optimal state.

### Ensemble FBA

To analyze the potentiality of a cell to explore alternative metabolic behaviors by altering its fluxes, we previously introduced the Ensemble Evolutionary FBA (eeFBA): [11] an extension of FBA defined with a goal to investigate putative flux distributions that can give rise to a specific metabolic behavior. With eeFBA, analyses are performed by generating a set of OFs where both terms and coefficients are selected randomly. Random OFs are subsequently optimized by means of linear programming and the computed flux distributions are filtered on the basis of one or more metabolic phenotypes definitions, to retrieve ensembles of solutions that are in agreement with the defined phenotypes.

### Retrieving kinetic parameters form a mechanism-based ensemble approach

Due to lack of information on kinetic constants, either with FBA or eeFBA it is not possible to determine metabolic concentrations at steady state. To overcome this limitation, in a recent paper [12], we proposed a strategy, where ensembles of phenotypes are still populated according to flux properties, but steady states are retrieved from mechanism based simulations. The parameters of the kinetic model are set using initial concentrations from the literature (whenever possible) and values for kinetic constants have been sampled randomly from a given interval (e.g. from 0 to the thermodynamic limit) thereby avoiding biases in their definition (see “Methods” section for further information).

With the above described procedure, we have been able to determine steady state metabolic concentrations that satisfy the definition of a given metabolic response to changing experimental conditions. It is worth to underline that, this readout was obtained without defining an OF, thus avoiding the assumption that the cell is performing an optimization towards a certain objective.

In this work we slightly modify the approach in order to take into account the fact that kinetic constants may assume different values under various experimental conditions due to enzymatic regulation. In order to associate a set of specific rate constants to the phenotype associated to each experimental condition, the kinetic constants are retrieved from a set of parameterizations of a mechanistic model that, when dynamically simulated with those constants, is able to generate time courses in agreement with the phenotype definition.

Strikingly, our method can be used to predict ensembles of rate constants that are in agreement with a given metabolic phenotype of interest only by providing its definition and a flux distribution for the same condition, obtained by means of FBA.

### EColiCore2: a case study

In [12], we applied our procedure exploiting a toy metabolic model of *S. cerevisie* and filtering trajectories accordingly to a definition of the Crabtree phenotype. In the present paper, we aim at investigating a more realistic metabolic reconstruction focusing on *Escherichia coli*, the prokaryotic model organism for which a number of core models have been built in a bottom-up fashion and are currently retrievable from the literature. A notable example, due to its wide exploitation, is the *E. coli* core model illustrated in Orth et al. [13].

Conversely, there is a relative scarcity of top-down metabolic models built starting from genome scale reconstructions of these bacteria. The EColiCore1 reconstruction was manually derived from the iAF1260 [14] genome scale model. However, this model has mainly testing and training purposes and is not completely consistent with the corresponding genome wide model.

Starting from the genome wide model iJO1366 [15], Hädicke et al. in [16] aimed at reconstructing a metabolic model of the central metabolism of *E. coli* called EColiCore2. This model, built with the final goal of establishing a reference core model for *E. coli* constraint-based analyses, has been derived reducing redundancies in biosynthetic routes and maintaining the degrees of freedom in the central metabolism, moreover, this core model is completely consistent with its genome wide counterpart. One key aspect of EColiCore2 is its ability to reproduce pivotal features of iJO1366, achieving a notable complexity reduction without losing its ability to depict emerging behaviors of *E. coli* metabolism.

**Fig. 1:**
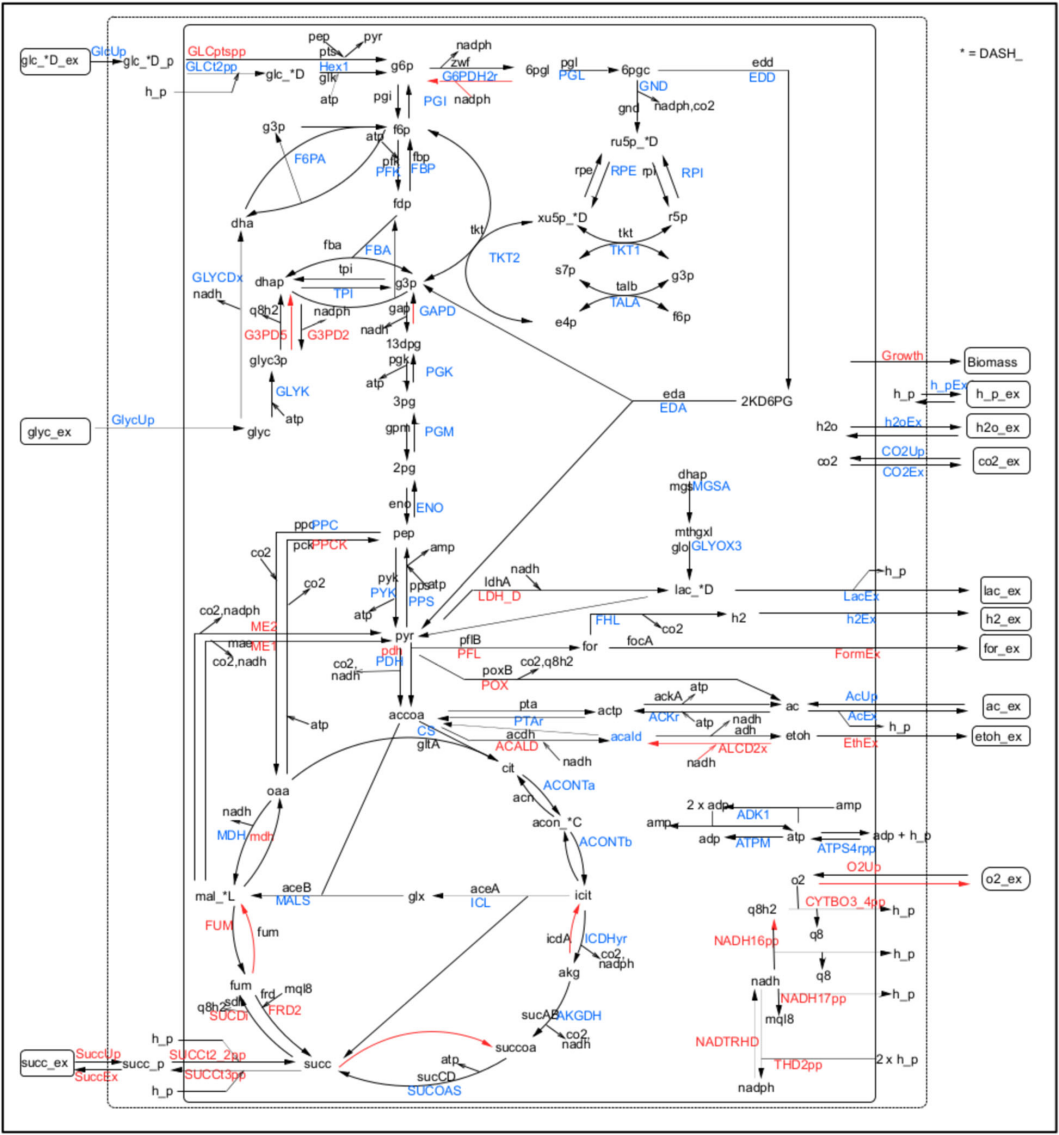
Wiring diagram of the EColiCore2 model. Metabolic network is modified (adding reverse reactions and cofactors) from Hädicke et al. [16]. In the map, reaction names are labeled in blue and placed next to the corresponding edge. The external environment is represented by a dashed contour, the cell is delimited by a solid contour. Significant reactions emerging from the Kolmogorov-Smirnov test described in section Results are labeled in red

ECC2comp, presented in [16] and illustrated in Fig. 1, is a further reduction of EColiCore2 derived by exploiting NetworkReducer [17], i.e., an algorithm able to automatically compress metabolic models by lumping linear chains of reactions in a single cumulative equation and by removing elements (metabolites and reactions) that are non essential to represent key metabolic functions referred to as “protected functions”.

## Methods

**Fig. 2:**
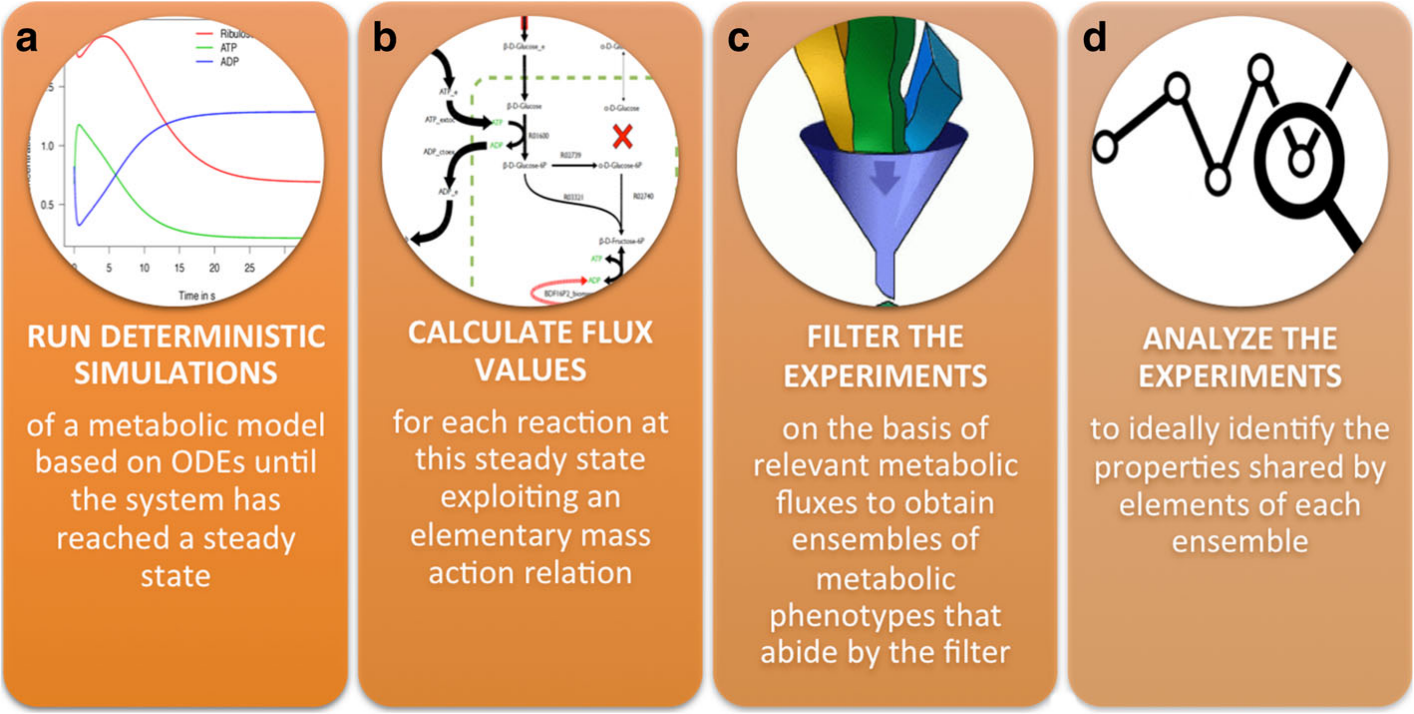
Schematic workflow illustrating the four main phases of the computational procedure. **a** Run deterministic simulations; **b** Calculation of flux values; **c** Filtering of experiments; **d** Analysis of outcomes. See main text for a complete description of the approach

The procedure introduced in [12] and schematically represented in Fig. 2 has been used here to setup the “experiments” hereafter illustrated. For a large number *P* of parametrizations we extract each of the M distinct kinetic constants of the model $\left (\vec {k}_{p} = \left (k^{1}_{p}, \ldots, k^{M}_{p}\right)\right)$ from a uniform distribution in [ 0,100). Every parametrization of this set is exploited to produce different simulations in accordance with each metabolic phenotype under study. We call metabolic phenotype a set of values assumed by key fluxes in defined experimental conditions. The mechanism based simulations, one for each different experimental condition, is performed using constant concentrations of the associated nutrient (e.g., glucose), oxygenation level and ions.

### Running deterministic simulations

To perform mechanism based simulations we assume mass-action kinetics within the ODEs deterministic framework. The metabolic model has been simulated until the achievement of a steady state for internal metabolites concentrations (Fig. 2a). Every simulation is considered at steady state at its ending time *t*_*e*_ if

1 $$ \frac{\sum^{M}_{w=1} \sigma\left(\left[\chi_{w}\right](\bar{t}, t_{e})\right)}{M - S} < \theta  $$

where $\sigma ([\!\chi _{w}](\bar {t}, t_{e}))$ is the standard deviation of the concentration [ *χ*_*w*_] of species *w* computed over $t_{e} - \bar {t}$ with $\bar {t} = 0.9\ t_{e}$ and *S* the number of species kept constant throughout the simulation and *θ* is a tolerance threshold.

### Calculating flux values

Afterwards, fluxes values *v*_*i*_ are calculated for every reaction (when the dynamic reaches the steady state) by means of the relation expressed by the mass action law illustrated in Eq. (2).

2 $$  v_{i} = k_{i} \prod_{w=1}^{M} [\!\chi_{w}]^{\alpha_{w i}}  $$

where *k*_*i*_ is the rate constant of reaction *i*, [*χ*_*w*_] is the concentration of species *w* and *α*_*wi*_ the stoichiometric coefficient with which species *w* participates in reaction *i* (Fig. 2b).

### Filtering the experiments

Once flux values have been obtained, experiments are filtered exploiting key metabolic fluxes in order to populate ensembles of metabolic phenotypes that are in agreement with the filter definition (Fig. 2c). In particular, in this work, to filter the experiments we defined 5 different phenotypes based on FBA simulations presented in [16].

### Analyzing the experiments

Finally, it is possible to analyze (Fig. 2d) the experiments to identify properties shared by elements of each ensemble, e.g., by investigating the presence of putative subphenotypes or by evaluating which reactions exhibit kinetic constants whose value depart from the average.

### *E. coli* case study

To test the procedure herein described we defined 5 different metabolic phenotypes (“protected phenotypes” in [16]) built on the basis of both the nutrient supplied and the oxygenation state observed (Table 1): exp1 - aerobic growth on glucose, exp2 - anaerobic growth on glucose, exp3 - aerobic growth on acetate, exp4 - aerobic growth on succinate, exp5 - aerobic growth on glycerol.

**Table 1:** Protected phenotypes. Phenotypes and maximal growth rate in the core model ECC2 obtained with FBA

ID	Description	Reached *μ* (ECC2)
exp1	aerobic growth on glucose	0.982
exp2	anaerobic growth on glucose	0.289
exp3	aerobic growth on acetate	0.244
exp4	aerobic growth on succinate	0.492
exp5	aerobic growth on glycerol	0.563

To evaluate the effectiveness of the procedure in discriminating the 5 phenotypes and in selecting corresponding ensembles of kinetic constants and steady state metabolic concentrations, we used ECC2comp [16]. We split the reversible reactions of the original compressed “core” into backward and forward reaction, obtaining a total of 114 irreversible reactions and 93 metabolites, of which 60 are internal, while 33 are external. The final model used in this study is provided in Additional file 1.

To determine the initial concentrations of metabolites involved in the *E. coli* model, we set them accordingly to the average values illustrated in the *E. coli* Metabolome Database (ECMDB) [18], an expertly curated database containing extensive metabolomic data and metabolic pathway diagrams about *Escherichia coli* (strain K12, MG1655). The ECMDB contains 3755 entries for metabolites and small molecules manually compiled including identification, taxonomy, concentrations, spectra, physical and biological properties. Information are derived from “original” data and from metabolic reconstructions, scientific articles, textbooks and other electronic databases. For metabolites in the model not having a concentration in ECMDB, we used the average value calculated over other retrieved values. The set of metabolic concentrations is provided in Additional file 2.

Metabolic phenotypes defined in this section need to be translated using a mathematical formalism in order to unequivocally characterize them as described in the following section. To this end we evaluated fluxes that in the ECC2comp model are proxies for the 5 phenotypes listed in Table 1.

### Populating the ensembles

To perform the procedure illustrated in this section, we implemented a set of scripts in plain vanilla Python available on GitHub (see Additional file 3). As already mentioned, dynamic simulations of the *E. coli* “core” metabolic model (step Fig. 2a) have been performed until reaching of the steady state exploiting a set of ordinary differential equations (ODEs) determined under the mass action kinetic assumption. The numerical integration of the ODEs system has been realized exploiting the software library LSODA (Livermore solver for ODEs with automatic method) [19] efficiently implemented in SciPy [20].

Due to a large volume of data produced with simulations (stored on GitHub, see Additional file 3), we decided to separate data generation and analysis phases. An efficient way to organize and access simulation outputs is to store them in a database. In particular here we exploited PyTables [21], a package for managing hierarchical datasets designed to efficiently and easily cope with extremely large amounts of data. PyTables makes use of the NumPy package and of the HDF5 library under the Python language.

Ensembles of kinetic constants sustaining the 5 different metabolic phenotypes have been populated by performing a large number of “experiments” conducted first by randomly defining, for each of them, the set of kinetic constants and then by executing a simulation for each given experimental condition providing, for each of them, a unique nutrient selected among glucose (exp1 and exp2), acetate (exp3), succinate (exp4), glycerol (exp5) and a non limiting amount of oxygen.

To populate the ensembles of kinetic constants, we filtered the experimental dataset according to conditions that have been implemented on the basis of fluxes illustrated in Table 2. The flux values in the table have been obtained by simulating the ECC2comp model under the 5 different experimental conditions (see Table 1) with FBA.

**Table 2:** Flux values used to set up filters in order to populate the 5 ensembles of kinetic constants corresponding to experimental conditions

	exp1	exp2	exp3	exp4	exp5
G6PDH2r	**4.142**	0.000	0.000	0.000	0.000
O2Up	*17.587*	0.000	*9.451*	*13.735*	*10.699*
GlycUp	0.000	0.000	0.000	0.000	*10.000*
MALS	0.000	0.000	**2.627**	0.000	0.000
AcEx	0.000	**7.835**	0.000	0.000	0.000
GND	**4.142**	0.000	0.000	0.000	0.000
SUCCt2_2pp	0.000	0.000	0.000	*10.000*	0.000
PGL	**4.142**	0.000	0.000	0.000	0.000
F6PA	0.000	0.000	0.000	0.000	**4.586**
SuccUp	0.000	0.000	0.000	*10.000*	0.000
GLYCDx	0.000	0.000	0.000	0.000	**4.586**
ALCD2x	0.000	**7.806**	0.000	0.000	0.000
ICL	0.000	0.000	**2.627**	0.000	0.000
EthEx	0.000	**7.806**	0.000	0.000	0.000
GLCptspp	*10.000*	*10.000*	0.000	0.000	0.000
AcUp	0.000	0.000	**10.000**	0.000	0.000
GlcUp	*10.000*	*10.000*	0.000	0.000	0.000
ME2	0.000	0.000	0.000	**3.488**	0.000
GLYK	0.000	0.000	0.000	0.000	**5.414**

Target fluxes have been calculated by means of FBA experiments. Reactions having non zero value in only one experimental condition are in bold; reactions defining the experimental condition (i.e., specific nutrients and oxygenation state) are in italic

In particular, to build filters we evaluated only ECC2comp reactions: (A) having non zero flux value in just one of the experimental conditions (Table 2, in bold), (B) defining the experimental conditions and oxygenation state (Table 2, in italic). For example, as the reaction GLYK is active only in metabolic phenotype exp5, flux distribution is assigned to metabolic phenotype exp5 iff the GLYK flux is greater than zero. Along similar lines O2Up, which defines the oxidation state, is active in every metabolic phenotype except exp2. Its flux distribution is assigned to metabolic phenotype exp2 iff the O2Up flux is equal to zero.

Formally these constraints relative to the phenotypes are summarized by logical expressions shown in Eqs. (3) to (6) where *v*_*i*_ represent the metabolic flux through the *i* reaction.

3 $$ {{}\begin{aligned} \mathbf{exp1:} \left({ v_{G6PDH2r}} > 0 \right) &\wedge \left({ v_{GND}} > 0 \right) \wedge \left({ v_{PGL}} > 0 \right)\\ & \wedge \left({ v_{GLCptspp}} > 0 \right) \wedge \left({ v_{O2Up}} > 0\right) \end{aligned}}  $$

4 $$ {\begin{aligned} \mathbf{exp2:} \left({ v_{AcEx}} > 0 \right) &\wedge \left({ v_{ALCD2x}} > 0\right) \wedge \left({ v_{EthEx}} > 0 \right) \\ &\wedge \left({ v_{GLCptspp}} > 0 \right) \wedge \left({ v_{O2Up}} = 0 \right) \end{aligned}}  $$

5 $$ {\begin{aligned} \mathbf{exp3:} \left({ v_{MALS}} > 0 \right) \wedge \left({ v_{ICL}} > 0 \right) &\wedge \left({ v_{AcUp}} > 0 \right) \wedge \left({ v_{O2Up}} > 0\right) \end{aligned}}  $$

6 $$ {\begin{aligned} \mathbf{exp4:} \left({ v_{SUCCt2_{2}pp}} > 0\right) &\wedge \left({ v_{ME2}} > 0 \right) \wedge \left({ v_{O2Up}} > 0 \right) \end{aligned}}  $$

7 $$ {\begin{aligned} \mathbf{exp5:} \left({ v_{GLYK}} > 0\right) &\wedge \left({ v_{F6PA}} > 0 \right) \wedge \left({ v_{GLYCDx}} > 0 \right) \\ &\wedge \left({ v_{O2Up}} > 0 \right) \end{aligned}}  $$

An experimental set of kinetic constants is assigned to a given ensemble (metabolic phenotype) if and only if all the constraints associated to that phenotype are satisfied.

## Results

### Obtained ensembles

To test the procedure on the simplified *E. coli* model, we tossed multiple different random sets of kinetic constants, keeping the concentration of ions and exchanged species (i.e., ac_ex, ca2_ex, cl_ex, co2_ex cobalt2_ex, cu2_ex, fe2_ex, fe3_ex, for_ex, glc_DASH_D_c, glc_DASH_D_ex, glc_DASH_D_p, h_ex, h2_ex, h2o_ex, k_ex, mg2_ex, mn2_ex, mobd_ex, MTHTHF_ex, nh4_ex, ni2_ex, o2_ex, pi_ex, so4_ex, succ_ex and zn2_ex) constant throughout the simulation time of 100 seconds, defined accordingly to [8] in order to allow the metabolic steady state to be reached after a perturbation (e.g. a pulse of nutrient).

Every simulation is considered at steady state if *θ* < 0.1*%*. If the steady state is verified, the random parametrization is retained, otherwise is dropped. To obtain a dataset of 10^4^ random sets of kinetic constants, we performed a total of 11520 samplings, thereby discarding the 13.2% of performed simulations. The total computational time to produce the data set has been 1d 2h 20min to run ODEs simulations on a workstation (8 x CPU 3.8 GHz Intel Core i7, RAM 32 GB) and producing 20.3 GB of data.

The input of the filtering procedure has been a dataset composed of 5·10^4^ simulations, i.e. 10^4^ random sets of kinetic constants tested over 5 experimental conditions.

**Fig. 3:**
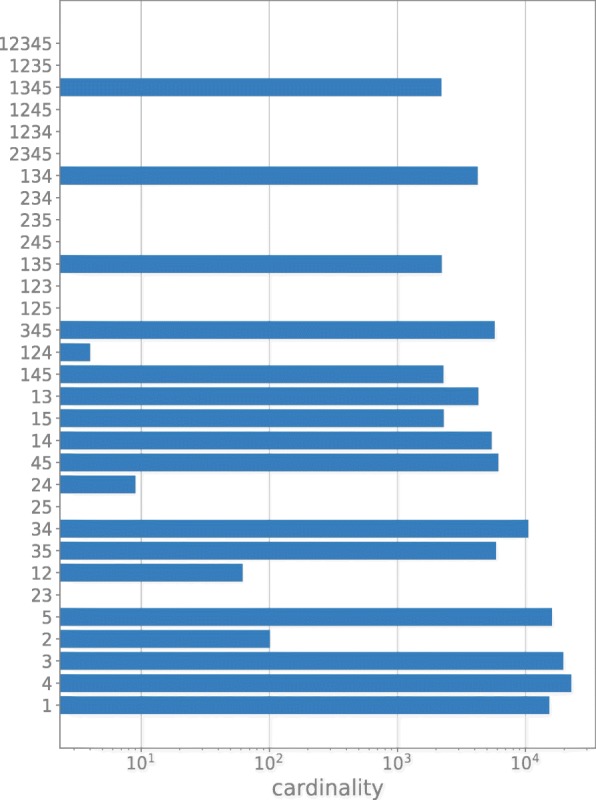
Cardinality of solutions illustrating the intersection among the different ensembles. Numbers on Y axis indicate the ensemble(s) (e.g. 12, indicates the ensemble exp1 and exp2) while the length of the bar indicates the number of solutions belonging to the ensemble or group of ensembles

We tamed numerical instability by imposing a threshold considering fluxes with a value less than 10^−10^ to be 0. From the dataset of 5·10^4^ solutions 15267 have been assigned to exp1, 101 to exp2, 19616 to exp3, 22719 to exp4 and 16033 to exp5, as illustrated by the last 6 rows of Fig. 3 reporting the cardinality of solutions, i.e. the number of random parameterizations that are assigned to one or more phenotypes at the same time.

Data presented in Fig. 3 show that we have been able to identify a subset of parametrization that work for all cases (1345 in Fig. 3) but not for the anaerobic condition (exp2 – 2 in Fig. 3). This reflects the consistent metabolic differences that can be pointed out in vivo between aerobic and anaerobic conditions.

**Fig. 4:**
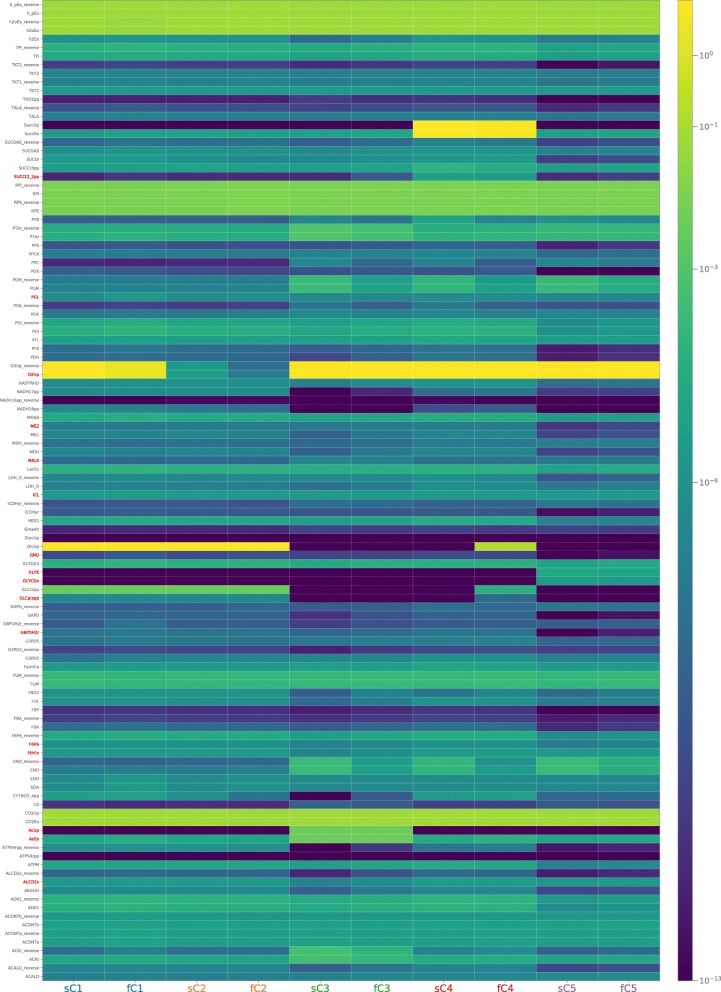
Heatmap. Figure illustrates median flux values through model reactions (rows) at the steady state, when the dynamic is labeled according to flux values at steady state emerging from their parameterization (columns labeled with sC#) and when it is filtered according to phenotypes (columns labeled with fC#). Red labels indicate reactions used to implement the filtering conditions for the metabolic phenotypes

In connection to this issue, we noticed that combinations exp2 - exp3 (23 in Fig. 3) and exp2 - exp5 (25 in Fig. 3) are empty sets due to the fact that in phenotype exp2 (anaerobic) reactions sustaining respiration are blocked (e.g. in TCA cycle the flux for reaction CS, leading to citrate is almost zero – see Fig. 4) while in exp3 and exp5 (aerobic conditions) the same reactions are active.

To compare flux values at steady state for each reaction in the system before and after the filtering, we drew the heatmap of Fig. 4 where rows list reactions, columns list sets of dynamics ensembles associated with each metabolic phenotype and the color represents the median value of that ensemble for that reaction at steady state (range [ 1·10^−13^, 1·10^1^]). We made two distinct associations of the dynamics to the phenotypes, the columns labeled as sC# have the dynamics assigned according to flux values at steady state emerging from their parameterization, whereas columns labeled as fC#, the filtered ones, are populated with the dynamics that satisfies phenotypes constraints at steady state, disregarding their initial condition. Overall, it is possible to notice that flux values in sC# and fC# for a given phenotype exhibit almost always a comparable flux value, there with only few exceptions to this behavior (e.g. reactions: O2Up_reverse less active in the fC2; h2Ex, PGM, PGM_reverse, PGK less active in fC3, SUCCt2_2pp more active in fC3; GlcUp and GLCt2pp more active in fC4). Moreover, comparing the different phenotypes, it is possible to notice that exp5 (sC and fC5) has flux values dissimilar to the other 4 phenotypes.

**Fig. 5:**
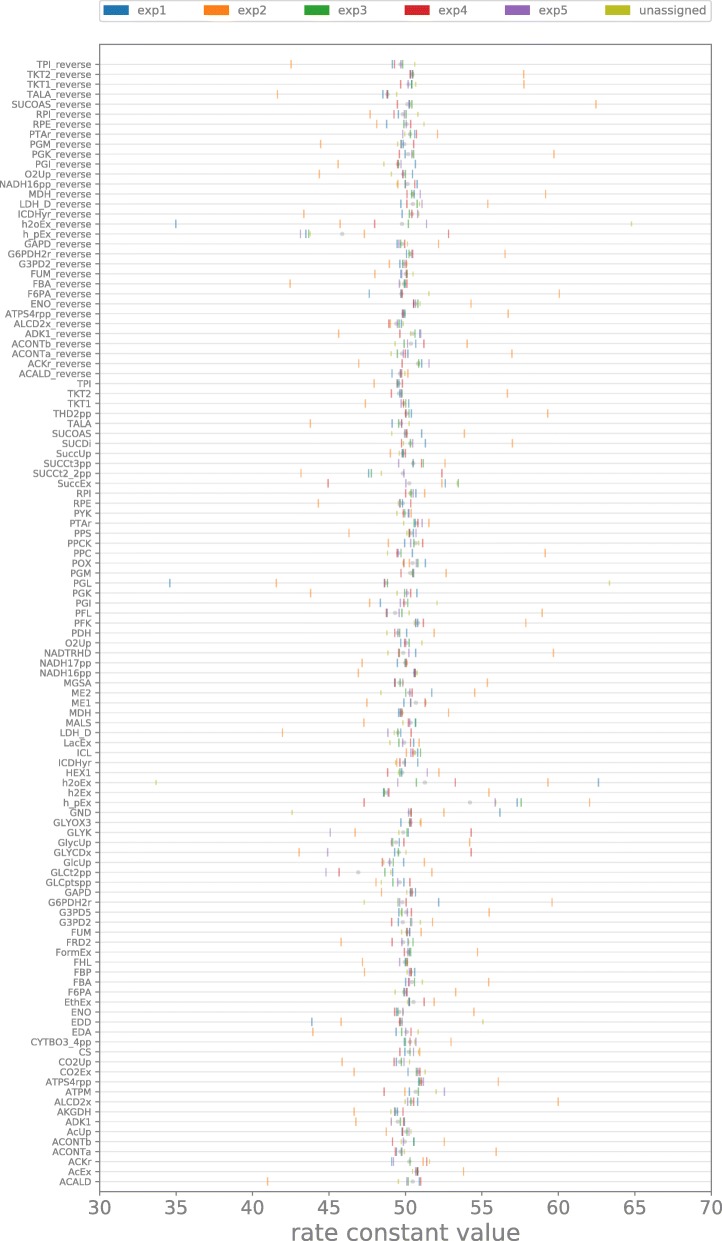
Boxplot. Illustration represents model reactions (rows), median for kinetic constants associated to the 5 phenotypes and not associated with any phenotype (colored vertical bars, see key for color code)

To better characterize the ensembles, we also plotted the median and the standard deviation for kinetic constants values retrieved for each ensemble after the filtering. Results illustrated in Fig. 5 show that there are little but non negligible differences in the median of kinetic constant values for all the reactions of the model (e.g. exp1 has different median values for h2o_Ex_reverse, F6PA_reverse, PGL, PGI, GND, h2o_Ex; exp5 has constant associated to ATPM greater than the average). Furthermore, supporting the findings that have emerged from analyzing cardinalities (Fig. 3), median values for the group of four aerobic phenotypes are very similar, while the medians for anaerobic phenotype are different form the previous group.

### Relevant fluxes

To identify relevant fluxes able to discriminate the 5 metabolic phenotypes, we performed a Kolmogorov-Smirnov (KS) test, a non-parametric hypothesis test procedure able to discern if two samples derive from the same distribution without investigating the actual shape of the distributions. The KS test has been performed for each possible pair of conditions and for each flux. The goal was to identify those fluxes that statistically differ for each pair of conditions (with significance 0.05) and that should thus be able to discriminate each of the 5 conditions.

From the output of the KS test we found a subset of 38 fluxes that can be regarded as relevant fluxes. Relevant fluxes are reported (red color) in Fig. 1. From their mapping on the metabolic network it emerges that these reactions are mainly part of functional elements in the network: in particular exchange reactions and hubs (i.e. network junctions connecting different pathways). Instead, reactions internal to pathways are less represented among the significant ones. This may suggest that the cell performs a tight regulation of fluxes among different pathways and a less stringent tuning for reactions belonging to the same pathway.

## Discussion

The analysis of average concentrations and relative standard deviations for molecular species during time courses shed light on some relevant issues hereafter discussed.

Overall, we underline that standard deviation values (*σ*) are small and few parameterizations (only 13% of the total) are discarded, suggesting that for the sampled interval [0, 1·10^2^] metabolism is robust towards kinetic constant variation. This parameter insensitivity has been further investigated in [22] where authors showed that many models in Systems Biology exhibit a “sloppy” spectrum of parameter sensitivities, concluding that besides the mere estimation of the parameter value, the community should focus on analyzing models in a predictive fashion.

**Fig. 6:**
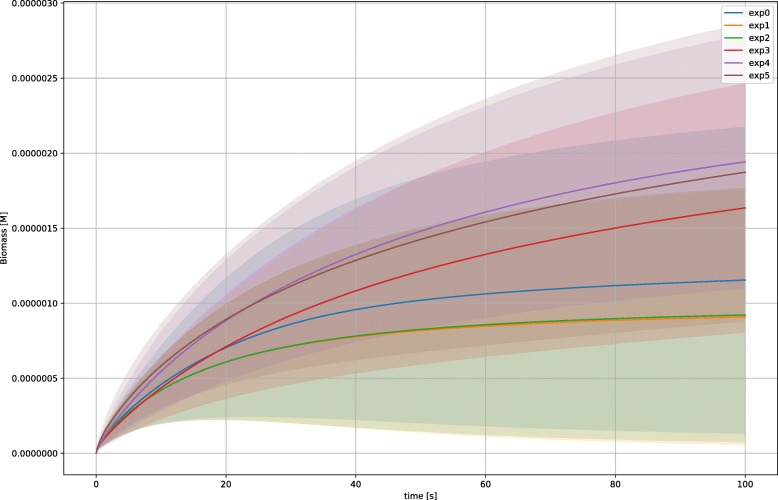
Time course for the species “biomass”. Figure shows that the mass of the system is accumulating during the simulation for every experimental condition, i.e., the system is able to grow under the experimental conditions. Shaded areas indicate the *σ* for every experiment, solid line represent a trajectory averaged over a subset of 200 parameterizations due to computational time limitations

Concerning biomass (Fig. 6) it is possible to notice that it is accumulating over time in all metabolic phenotypes. Interestingly, when we tested a further experimental condition (exp0 – not used as a metabolic phenotype) representing an enriched growth media (i.e., when all the nutrients are simultaneously available), this turned out not to be the condition leading to the maximal level of biomass (it is for instance the aerobic growth on succinate, exp4 – purple line).

**Fig. 7:**
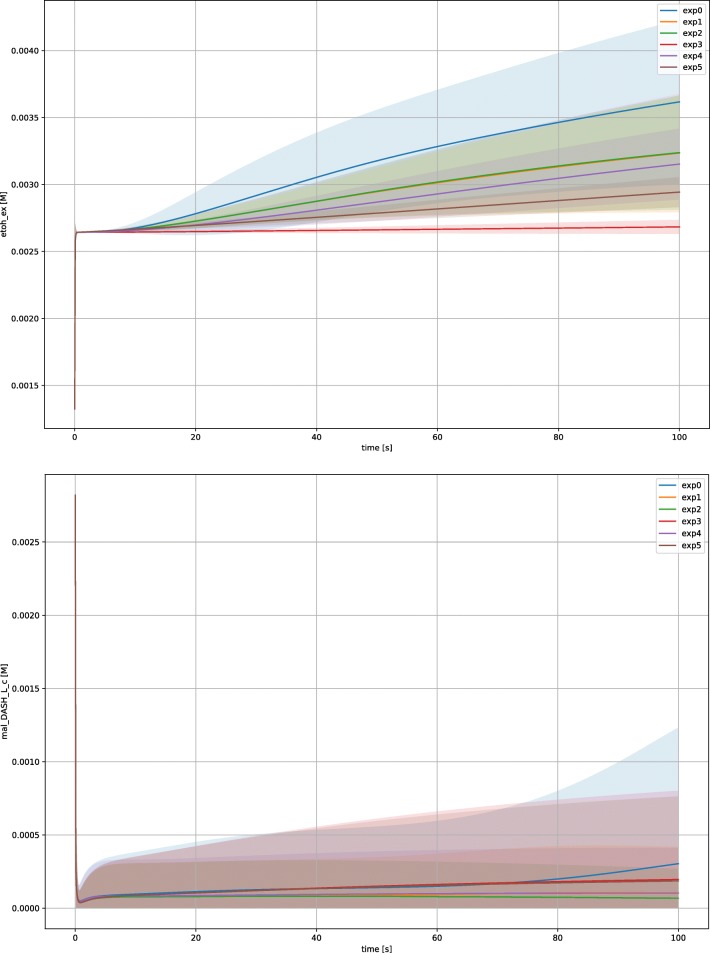
Time course for the species ethanol and malate. The time course for the species ethanol (top) shows that the species (not evaluated for the determination of the steady state) is accumulating during the simulation for every experimental condition, i.e., the system is able to perform alcoholic fermentation. Instead the time course for malate (bottom), shows the reaching of the steady state indicating that the system is also using the TCA cycle. Shaded areas indicate the *σ* for every experiment, solid line represent a trajectory averaged over a subset of 200 parametrizations due to computational time limitations

Furthermore, the analysis of time courses (Fig. 7) reveals that many metabolic pathways remain active throughout the simulation since they are generating metabolic intermediates. As an example *E. coli* is performing both alcoholic fermentation, as it appear from the time course for ethanol (Fig. 7 top), and TCA cycle, a pathway considered as indicator for respiration and illustrated at the bottom of Fig. 7, with the time course for malate.

**Fig. 8:**
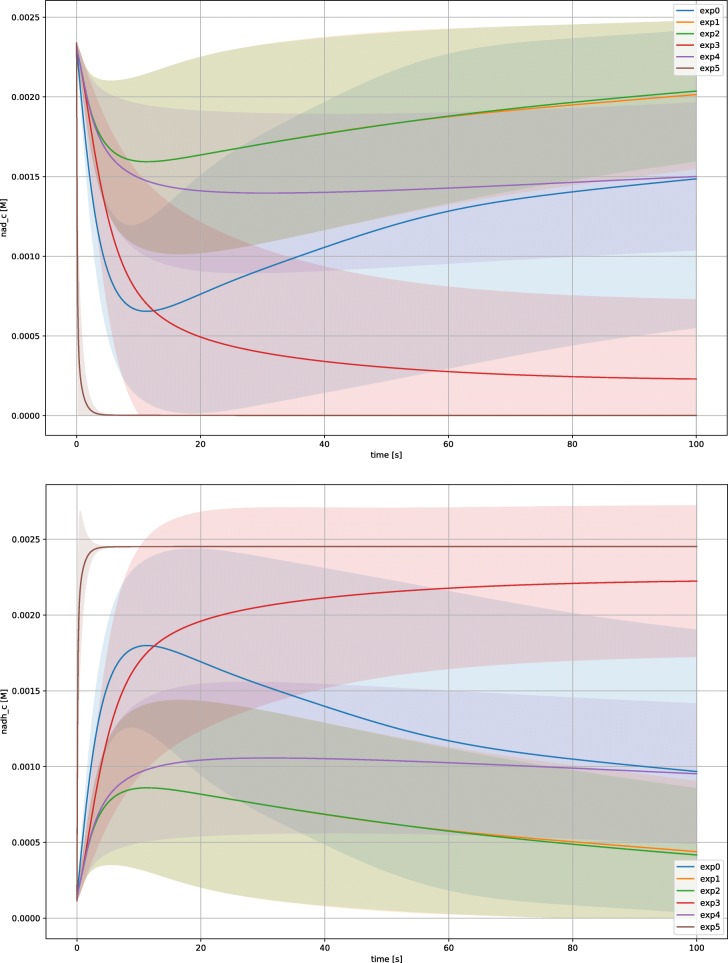
Time courses for the species NAD (top) and (NADH) bottom. Figure illustrate that the species are satisfying the steady state condition (i.e., are not varying more than 1% in the last 10 s of simulation. Moreover, NAD/NADH ratio is compatible with “sustained steady states” in all experimental condition except experiment 5. Similar time courses are obtained for NADP and NADPH. Shaded areas indicate the *σ* for every experiment, solid line represent a trajectory averaged over a subset of 200 parametrizations due to computational time limitations

Data supporting the actual activation of biochemical pathways in the model are also the presence of steady states for cofactors such as NAD/NADH and NADP/NADP which appear to be dynamically inter-converted as shown in Fig. 8 where, comparing the time courses for NAD (top) and NADH (bottom), it is possible to notice a symmetrical trend. This fact indicates that metabolic pathways are maintaining the system energetically active and capable of generating biomass. Focusing on the set of kinetic constants assigned to the different metabolic phenotypes, the procedure illustrated in the present paper led to the population of all the 5 phenotypes and to the identification of a subset of kinetic constants assignable to the four aerobic conditions. Unfortunately, there is no single “universal” parametrization assignable to all 5 phenotypes. This fact could be determined by different causes such as an under sampling of random kinetic constants, a too narrow sampling interval (in this study 2 orders of magnitude), or an excessively relaxed filtering condition not allowing a complete discrimination among the phenotypes.

Furthermore, the evaluation of median and standard deviation for the kinetic constants belonging to the 5 ensembles (Figs. 4 and 5) suggests that there are only few reactions that have to be finely tuned in order to direct the system towards a specific metabolic phenotype, a fact that suggests once more that metabolism is a system particularly robust towards perturbations. In this case a global sensitivity analysis would help to investigate the issue of robustness more specifically.

## Conclusions

Constraint-based models have been successfully implemented to study metabolic fluxes at steady state, nevertheless, information about the temporal evolution of the system during the transient phase preceding the steady state can not be derived from them. In addition, the metabolic concentrations at steady state can not be deduced from constraint-based methods since there is no information about kinetic constants.

Computational approaches developed in [12] and exploited in the present work are an improvement designed to override limitations by exploiting mechanism-based simulations. Here, initial conditions are partially retrieved from the literature (molecular concentrations) and kinetic constants are randomly determined. Figure 3 sums up the readout of the procedure: through a filtering procedure based on a loose definition of the 5 experimental conditions (metabolic phenotypes) involving some key reactions, the developed method is able to assign random sets of kinetic constants to one or more metabolic phenotypes.

With the present contribution we aimed at improving and testing a computational framework capable of retrieving ensembles of kinetic constants that can be associated with different metabolic phenotypes. It is worth underlying that, in contrast with constraint-base approaches, our method is not assuming that metabolism is optimized to perform a specific task.

We underline that the methodology used here can be exploited to retrieve ensembles of kinetic constants for any metabolic phenotype providing only its formal definition (in terms of nutrients supplied and oxygenation state together with an estimation of initial concentrations for modeled species) and a flux distribution obtained by means of a constraint-based simulation (for which no kinetic parameters are needed).

For what concerns perspectives, we plan to better characterize the metabolic steady state by exploiting more efficient strategies to calculate whether the system reaches a stationary condition. Among these strategies a promising approach includes the use of the NLEQ2 non-linear root-finding algorithm [23]. Moreover, we are considering to significantly expand the sampled set of kinetic constants through a significant speed-up of simulations achieved by means of high performance and parallel computing applied to Systems Biology modeling problems [24, 25].

## Additional files

Additional file 1 ECC2C.xml. SBML file for the ECC2comp model of *E. coli* used for the analysis. (XML 69. 8 kb)

Additional file 2 X0etc.xlsx. In tab “conc” are listed initial concentrations of metabolites for the 5 different phenotypes. In tab “FeedNoFilt” are listed metabolites provided at constant concentration throughout the simulation and metabolites not evaluated to verify the steady state. (XLSX 14.8 kb)

Github. The generated dataset (ECcoliExpsParam_10_Filter_0.001.h5) and python scripts implemented for this study are deposited on a GitHub repository at http://github.com/riccardocolombo/kineticensemble (ZIP 1.52e+7 kb)

## Abbreviations

ECMDB: E. coli metabolome database
eeFBA: Ensemble evolutionary FBA
FBA: Flux balance analysis
KS: Kolmogorov-Smirnov LSODA: Livermore solver for ODEs with automatic method
ODEs: Ordinary differential equations
OF: Objective function
